# Species-dependent impact of immunosuppressive squalene-gusperimus nanoparticles and adipose-derived stem cells on isolated human and rat pancreatic islets

**DOI:** 10.1080/19382014.2022.2100191

**Published:** 2022-07-15

**Authors:** Carlos E. Navarro Chica, Tian Qin, Erika Pinheiro-Machado, Bart J. de Haan, M.M. Faas, Alexandra M. Smink, Ligia Sierra, Betty L. López, Paul de Vos

**Affiliations:** a Department of Pathology and Medical Biology, Section of Immunoendocrinology, University Medical Center Groningen, University of Groningen, Groningen, the Netherlands; b Grupo de Investigación Ciencia de los Materiales, Instituto de Química, Facultad de Ciencias Exactas y Naturales, Universidad de Antioquia, Medellín, Colombia

**Keywords:** Squalene-gusperimus nanoparticles, adipose-derived stromal cells (ASCs) immunosuppressant, gene expression, qRT-PCR, pancreatic islets

## Abstract

Transplantation of pancreatic islets is a promising approach to controlling glucose levels in type 1 diabetes mellitus (T1DM), but islet survival is still limited. To overcome this, islet co-culture with mesenchymal stromal cells (MSCs) together with safe immunosuppressive agents like squalene-gusperimus nanoparticles (Sq-GusNPs) may be applied. This could support islet survival and engraftment. Here, we studied how Sq-GusNPs and adipose-derived stem cells (ASCs) influence islets response under pro-inflammatory conditions. Through qRT-PCR, we studied the expression of specific genes at 24 hours in human and rat islets and ASCs in co-culture under indirect contact with or without treatment with Sq-GusNPs. We characterized how the response of islets and ASCs starts at molecular level before impaired viability or function is observed and how this response differs between species. Human islets and ASCs responses showed to be principally influenced by NF-κB activation, whereas rat islet and ASCs responses showed to be principally mediated by nitrosative stress. Rat islets showed tolerance to inflammatory conditions due to IL-1Ra secretion which was also observed in rat ASCs. Human islets induced the expression of cytokines and chemokines with pro-angiogenic, tissue repair, and anti-apoptotic properties in human ASCs under basal conditions. This expression was not inhibited by Sq-GusNPs. Our results showed a clear difference in the response elicited by human and rat islets and ASCs in front of an inflammatory stimulus and Sq-GusNPs. Our data support the use of ASCs and Sq-GusNP to facilitate engraftment of islets for T1DM treatment.

## Graphical abstract

**Figure:**
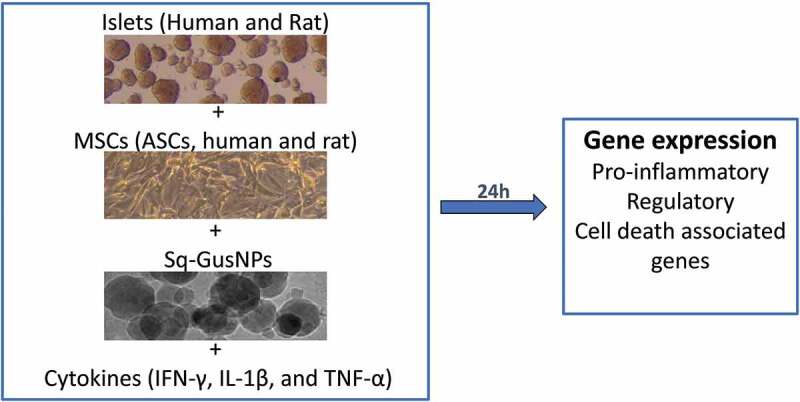

## Introduction

Transplantation of pancreatic islets is a promising alternative for exogenous insulin in patients with type 1 diabetes mellitus (T1DM). The advantage of islet transplantation over other therapies is that it regulates glucose on a minute-to-minute level.^1^ This approach has reached a certain degree of success, but its broad application is hampered by the high variation in the survival rate of the islets after the grafting procedure. The high variation has been attributed to factors such as differences in islet quality but also sensitivity to hypoxia and low nutrient conditions after implantation as well as to the cellular and humoral response triggered after transplantation.^1^,^2^ To overcome this, many strategies have been proposed to increase islet survival. Among these strategies is the use of new, safe immunosuppressive agents that are released in a controlled fashion to provide an anti-inflammatory microenvironment around islets in the transplantation site as well as the use of mesenchymal stromal cells that have shown to increase islets survival rate.^3–9^

Mesenchymal stromal cells (MSCs) are a somatic cell population present in many perinatal and adult tissues.^10^ They can be isolated from several tissues such as bone marrow, adipose tissue, connective tissue, umbilical cord, menstrual blood, and nasal mucosa.^11^ MSCs can self-renew, are easy to culture *in vitro*, and can differentiate into different cell types.^10^,^12^ These cells also have a high regenerative effect on surrounding tissue due to the capacity to produce soluble trophic factors.^13^ This can promote survival, regeneration, and function of different cells including those of pancreatic islets through paracrine mechanisms driven by MSCs-derived growth factors, cytokines, and chemokines.^9^,^14^ The factors secreted by MSCs can also modulate the immune system by inhibition of activation and proliferation of lymphocytes T and B, and dendritic cells, as well as by induction of polarization of macrophages from M1 to M2 state.^12^ Therefore, co-transplantation of pancreatic islets with MSCs is considered to have a high potential to prolong functional graft survival.^9^

Although MSCs have been proposed as a regeneration-supporting and anti-inflammatory cell-based approach for pancreatic islet transplantation,^9^ so far it has not been studied whether MSCs maintain these functional traits when exposed to immunosuppressive conditions. This is relevant as currently, islet transplantation is in the vast majority of cases applied in patients that are receiving a kidney transplant because of end-stage renal failure and chronic immunosuppression.^15^ Also, new methodologies are currently developed to apply local immunosuppression and controlled release of drugs to manage inflammatory responses around the grafts but their effects on MSCs function co-transplanted with islets is unknown.^16–19^

During the isolation and transplant process islets are submitted to harsh conditions that reduce the functional mass of the graft limiting its success. Especially the immediate posttransplant period is an extremely delicate period in which more than 60% of the islets have been reported to die due to enhanced sensitivity to inflammatory stress.^20^ This enhanced sensitivity is caused by a combination of disruption of the interactions between the islets and the peri-insular extracellular matrix as well as interruption of direct vascular access during the isolation process leading to low nutrient supply, high oxidative and hypoxic stress, and significant loss of insulin-producing cells posttransplant.^14^ To lower this loss we presented in a previous study a new controlled release system to modulate immunity in the immediate period after implantation.^20^ This involves squalene-gusperimus nanoparticles (Sq-GusNPs), a nanoparticulate system designed to encapsulate the immunosuppressive drug gusperimus to enhance the efficacy of this powerful anti-inflammatory drug.^21^

Adipose-derived stem cells (ASCs) are often used as an MSCs source because they have shown superiority regarding the secretion of paracrine molecules and they can be relatively easily isolated from the patient itself by taking a fat biopsy.^14^ So, they can serve as an autologous treatment in islet transplantation. Additionally, Sq-GusNPs have been shown to maintain the viability and function of human pancreatic islets under inflammatory conditions without cytotoxic effects.^20^ Here, we co-cultured pancreatic islets with ASCs in the presence and absence of IFN-γ, IL-1β, and TNF-α. This was done to gain insight into how islets and ASCs mutually influence each other in the presence or absence of an immunosuppressive agent under inflammatory or basal conditions. We determined the gene expression of pro-inflammatory, regulatory cytokines, or cell-death associated genes in both islets and ASCs after stimulation and/or treatment in both human and rat islets and ASCs. With this, we aimed to characterize the inflammatory response in the islets and how ASCs are influenced by the inflammatory conditions at molecular level in the first 24 hours. Additionally, we studied the differences in expression profiles between rat and human cells and whether potential synergistic effects between ASCs and Sq-GusNPs occur that contribute to functional survival of pancreatic islets.

## Materials and methods

### Materials

The bioconjugate squalene-gusperimus (Sq-Gus) was obtained starting from squalene and through consecutive reaction steps until obtention of the squalene carbocyclic acid derivative which was further reacted with gusperimus as previously reported.^21^ Absolute ethanol (EtOH) and HEPES were purchased from Merck (Darmstadt, Germany). DMEM supplemented with 4.5 g/L glucose and L-Glutamine was purchased from LONZA (Walkersville, MD, USA). CMRL-1066, Glutamax, penicillin-streptomycin, L-Glutamine, alamarBlue^TM^ reagent, TRIzol^TM^ reagent, and SuperScript II Reverse Transcriptase were purchased from Life Technologies Europe BV (Bleiswijk, The Netherlands). Fetal calf serum (FCS), D-(+)-Glucose, FastStart Universal SYBR Green Master (Rox), Histopaque®-1119, Histopaque®-1077, DNase I Roche, Corning® Transwell® polyester membrane cell culture inserts pore size 0.4 μm, membrane diameter 12 mm, and human and rat primers associated with gene expression of different cytokines and inflammatory factors were purchased from Sigma-Aldrich Chemie N.V. (Zwijndrecht, The Netherlands). Recombinant human interferon-gamma (rh IFN-γ), recombinant human interleukin-1 beta (rh IL-1β), recombinant human tumor necrosis factor-alpha (rh TNF-α), recombinant rat interferon-gamma (rr IFN-γ), recombinant rat interleukin-1 beta (rr IL-1β), and recombinant rat tumor necrosis factor-alpha (rr TNF-α) were purchased from ImmunoTools (Friesoythe, Germany). Human insulin ELISA kit Mercodia was purchased from Bio-Connect Diagnostics B.V. (Te Huissen, The Netherlands). Ultra-sensitive Rat Insulin ELISA Kit was purchased from Crystal Chem Europe (Zaandam, The Netherlands). Collagenase NB4 Standard Grade was purchased from Nordmark Pharma GmbH (Uetersen, Germany). Collagenase NB8 was purchased from Serva Electrophoresis (Heidelberg, Germany). The Invitrogen™ Quant-iT™ PicoGreen™ dsDNA reagent was purchased from Fisher Scientific (Landsmeer, The Netherlands).

### Squalene-gusperimus nanoparticles (Sq-GusNPs) preparation

Sq-GusNPs were prepared through nanoprecipitation as previously reported.^21^ Briefly, the Sq-Gus bioconjugate was dissolved in EtOH at a concentration of 2 mg/mL. Later, 380 μL of the solution was added drop by drop to 1 mL of deionized water under stirring (500 rpm) for 10 minutes after which EtOH was evaporated using the concentrator SpeedVac SPD2010 (Thermo Fisher Scientific, Bleiswijk, The Netherlands). This resulted in an aqueous suspension of pure NPs with a size of 189.2 ± 73.83 nm as measured by dynamic light scattering (DLS) with the particle size analyzer NICOMP 380 ZLS (Particle Sizing Systems, Inc., Santa Barbara, CA, USA).

### Cell culture

Pancreatic human or rat islets were cultured in CMRL-1066 medium supplemented with 8.3 mM glucose, 20 mM HEPES, 10% FCS heat-inactivated, 2 mM Glutamax, and 50 μg/mL penicillin/streptomycin as previously reported.^22^ Adipose tissue-derived stem cells (ASCs) from human or rat were cultured in DMEM supplemented with 4.5 g/L glucose and L-Glutamine, 10% FCS heat-inactivated, L-glutamine (2 mM), and 50 μg/mL penicillin/streptomycin. ASCs and islets were cultured in an incubator at 37°C and 5% CO_2_.

### Islet isolation

#### Human islets

Human pancreatic islets were isolated from pancreata of deceased donors as previously described.^23^ The islets were obtained from the Leiden University Medical Center or through the JDRF award 31–2008-416 (European Consortium for Islet Transplantation, Islet for Basic Research program, Milan, Italy). Donor characteristics are specified in Table 1. Islets were used for research when the quality and/or number were insufficient for clinical application according to national laws and when research consent was available. All the procedures were approved and carried out under the code of proper secondary use of human tissue in The Netherlands as formulated by the Dutch Federation of Medical Scientific Societies. After shipment to the University Medical Center Groningen islets were cultured for 24 hours as described in the cell culture section before starting the experiments.

**Table 1.** Donor information for human islets.

*Donor*	*Age (years)*	*Gender*	*BMI (Kg/m^2^)*	*Islet isolation center*	*Death cause*	*Purity (%)*	*Viability (%)*
1	54	Male	35	LUMC^a^	Non-cardiac	55	>80
2	59	Male	23	ECIT^2^	Cerebral bleeding	80	95
3	74	Female	26	LUMC	Non-cardiac	75	>80
4	57	Female	27	ECIT	Cerebral bleeding	60	95
5	53	Female	27.2	ECIT	Cerebral bleeding	75	90

*^a^LUMC: Leiden University Medical Center;^2^ECIT: European Consortium for Islet Transplantation*

#### Rat islets

The Dutch Central Committee on Animal Testing (CCD) and Animal Welfare Authority at the University of Groningen approved all described animal procedures (CCD project number: AVD1050020185726). All experiments and procedures were performed in accordance with the Institution Animal Care Committee of the University of Groningen. All animals received animal care in compliance with the Dutch law on experiment animal care. Rat pancreatic islets were isolated from different and independent groups of Sprague-Dawley rats (Envigo, Horst, The Netherlands) weighing 250–300 g as previously described.^24^,^25^ Briefly, under anesthesia, the pancreas was distended by injecting a solution of collagenase NB8/DNase I into the bile duct. After dissection, the tissue was further digested by incubating the distended pancreas for 18 min in a water bath at 37°C under shaking. Subsequently, islets were separated from the exocrine tissue by a Histopaque (1119, 1077) density gradient. Islets with a diameter of 30–150 µm were handpicked, counted, and cultured for 24 hours as described in the cell culture section before starting the experiments.

### Adipose tissue-derived stem cells (ASCs) isolation from perirenal fat

Human perirenal fat was obtained from surgical waste material of healthy kidney donors. For this purpose, no informed consent is required according to guidelines approved by University Medical Center Groningen (UMCG). Rat perirenal fat was isolated from Sprague-Dawley rats. Perirenal fat tissue was transferred to a petri dish and 2 mL of Hank’s balanced salt solution buffer (HBSS) was added. After cutting the tissue into small pieces, it was transferred to a 50 mL conical tube with 10 mL of HBSS and centrifuged at 500 ×*g* for 5 minutes at room temperature. Subsequently, HBSS solution was removed, washed again with additional 10 mL of HBSS, centrifuged at 500 ×*g* for 5 minutes at room temperature, and HBSS solution removed. Then, 0.5 mg/mL collagenase NB4 solution in DMEM supplemented with 1% penicillin/streptomycin was added to the fat and incubated for 30 minutes in a water bath at 37°C under shaking. Next, the mixture was vortexed and to stop digestion we added 20 mL of DMEM supplemented with 10% FCS, 1% penicillin/streptomycin, and 1% glutamine. The tube was centrifuged at 700 ×*g* for 7 minutes at room temperature, and the content washed again with 20 mL DMEM supplemented with 10% FCS, 1% penicillin/streptomycin, and 1% glutamine. Finally, cells were centrifuged at 700 ×*g* for 7 minutes at room temperature, fat and medium were removed, and ASCs in the pellet were transferred to a culture flask and cultured as indicated in cell culture section until use. The passage number of ASCs used in the experiments was 1–6. ASCs were seeded 24 h before starting the experiments.

### Cell viability with alamarBlue^TM^

Cell viability was determined using the alamarBlue^TM^ reagent as previously reported.^20^ Briefly, the reagent was diluted in culture medium (10% v/v). After treatment, ASCs or islets were washed with PBS and incubated for four hours with 0.5 mL of the diluted reagent. Later, fluorescence was measured with the plate reader CLARIOstarPlus (BMG LABTECH, Offenburg, Germany) Ex/Em 560/590 nm. For the islets, results were normalized to total DNA content as was determined using the Quanti-iT PicoGreen dsDNA kit (Invitrogen). Fluorescence obtained from ASCs or islets without any treatment was used as reference and control. Results were expressed as percentage of the control.

### Glucose-stimulated insulin secretion (GSIS) test

After treatment, 25 handpicked islets were washed 2 times with 2.75 mM glucose solution prepared in 25 mM KRH buffer (low glucose solution). After a 90 minutes preincubation period (human islets) or 30 minutes (rat islets) with low glucose solution, islets were incubated for 60 min (human islets) or 45 min (rat islets) with low glucose solution, followed by a 60 min (human islets) or 45 min (rat islets) incubation with a 16.5 mM glucose solution prepared in 25 mM KRH buffer (high glucose solution). Finally, islets were washed a single time with low glucose solution and incubated for 60 min (human islets) or 45 min (rat islets) with low glucose solution. From each incubation step, supernatants were collected, and insulin secretion was determined using the insulin ELISA kit Mercodia for human islets or Crystal Chem for rat islets. Results were normalized to total DNA content as was determined using the Quanti-iT PicoGreen dsDNA kit (Invitrogen). The stimulation index (SI) was calculated by dividing the amount of insulin secreted after incubation with the high glucose solution by the amount secreted after the first incubation with low glucose solution.

### Insulin ELISA

Sandwich ELISA for human insulin (Mercodia) and rat insulin (Crystal Chem) were performed according to the manufacturer’s instructions using a microplate spectrophotometer Benchmark Plus BIO-RAD (Bio-Rad Laboratories B.V., Veenendaal, The Netherlands) at 450 nm with correction at 630 nm for the rat insulin assay.

### Effect of treatment with Sq-GusNPs, ASCs, and inflammatory cytokines on viability and function of pancreatic islets

To determine how Sq-GusNPs, ASCs, and inflammatory cytokines influence viability or function of pancreatic islets, 40 handpicked islets were cultured alone or cocultured with ASCs (50.000 ASCs/mL) without direct contact in presence or absence of Sq-GusNPs at a concentration of 22.31 µg/mL or a blend of proinflammatory cytokines (IFN-γ 2000 U/mL, IL-1β 150 U/mL, and TNF-α 2000 U/mL) in 24 well Transwell plates in a total volume of 1 mL of the culture medium used for islet culture. After 24 hours incubation, viability of islets and ASCs was determined with the alamarBlue^TM^ reagent, and islet function was tested through GSIS. ASCs and islets without treatment were used as control group.

### Quantitative reverse-transcription polymerase chain reaction (qRT-PCR)

A qRT-PCR was performed to determine the expression of genes associated with the secretion of different cytokines and factors associated with an inflammatory response. After treatment, islets or ASCs were homogenized with TRIzol^TM^ reagent, and total RNA was isolated following the manufacturer’s instructions. Later cDNA was synthesized using SuperScript II Reverse Transcriptase. Finally, qPCR was performed with a FastStart Universal SYBR Green Master for the genes associated with the secretion of different cytokines (primer sequences are listed in Tables 2 and 3). Reactions were carried out in 384-well PCR plates (Thermo Scientific) using a ViiA7 Real-Time PCR System (Applied Biosystems, Carlsbad, CA, USA). Delta Ct (ΔCt) values were calculated and normalized to the housekeeping genes GAPDH, and β-actin. The 2^−(ΔΔC^_T_^)^ method was used for the comparative quantification of gene expression.^26^

**Table 2.** Human primer sequences used for qRT-PCR.

Gene	Forward sequence 5’ – 3’	Reverse sequence 5’ – 3’
GAPDH	CAAATTCCATGGCACCGTCAA	AGCATCGCCCCACTTGATTT
β-actin	CGCGAGAAGATGACCCAGAT	AGCACAGCCTGGATAGCAAC
TNF-α	GAGGCCAAGCCCTGGTATG	CGGGCCGATTGATCTCAGC
IL-8	ACTCCAAACCTTTCCACCCC	TTCTCAGCCCTCTTCAAAAACTTC
IL-6	ACTCACCTCTTCAGAACGAATTG	CCATCTTTGGAAGGTTCAGGTTG
IL-1β	AGCTACGAATCTCCGACCAC	CGTTATCCCATGTGTCGAAGAA
IL-10	TCAAGGCGCATGTGAACTCC	GATGTCAAACTCACTCATGGCT
IL-4	ACAGCCTCACAGAGCAGAAGACT	TGTTCTTGGAGGCAGCAAAGA
IL-13	CCTCATGGCGCTTTTGTTGAC	TCTGGTTCTGGGTGATGTTGA
IL-1Ra	CAATGCTGACTCAAAGGAGACGA	TCCCTCCATGGATTCCCAAGA
NF-κB (p50)	GCAGCACTACTTCTTGACCACC	TCTGCTCCTGAGCATTGACGTC
iNOS	GCCACAGAAGAGCCTGAGAG	GATCTCTGTGGGCGTGTGAT
MCP-1	GAAAGTCTCTGCCGCCCTT	GGTGACTGGGGCATTGATTG
IP-10	CTGTACGCTGTACCTGCATCA	TGATGGCCTTCGATTCTGGA
IL-15	AACAGAAGCCAACTGGGTGAATG	CTCCAAGAGAAAGCACTTCATTGC
RIPK1	AGCTCCTGGGCGTCATCATA	AGGTCTGCGATCTCGGCTTT
RIPK3	TAATGTGGGCAGTGCTTGCT	GTCTGTCCTTGGGCTCACTG
BAD	CCCAGAGTTTGAGCCGAGTG	CCCATCCCTTCGTCGTCCT
BID	GGCCTACCCTAGAGACATGGA	AGACATCACGGAGCAAGGAC
Caspase 3	CATACTCCACAGCACCTGGTT	TCAAGCTTGTCGGCATACTGT
Caspase 9	TTTGAGGACCTTCGACCAGC	CGAAACAGCATTAGCGACCC

**Table 3.** Rat primer sequences used for qRT-PCR.

Gene	Forward sequence 5’–3’	Reverse sequence 5’–3’
GAPDH	AAGGTCGGTGTGAACGGATTT	CTTTGTCACAAGAGAAGGCAGC
β-actin	GAACCCTAAGGCCAACCGTGAA	TACGTACATGGCTGGGGTGT
TNF-α	CATCAAGAGCCCTTGCCCTA	CTGGAAGACTCCTCCCAGGTA
CXCL-1	CCACACTCAAGAATGGTCGC	ACTTGGGGACACCCTTTAGC
IL-6	CCTACCCCAACTTCCAATGCT	ATGGTCTTGGTCCTTAGCCAC
IL-1β	CAGGATGAGGACCCAAGCAC	GTCGTCATCATCCCACGAGT
IL-10	GGGAGAGAAGCTGAAGACCC	ATTCATGGCCTTGTAGACACCT
IL-4	TGTAGAGGTGTCAGCGGTCT	TCAGTGTTGTGAGCGTGGAC
IL-13	AGTTGCAATGCCATCCACAG	CCACATCCGAGGCCTTTTGG
IL-1Ra	GGGACCTTACAGTCACCTAATCTC	GGTCTTTTCCCAGCAGGGTG
NF-κB (p65)	ATCAATGGCTACACGGGACC	AGTTCATGTGGATGAGGCCG
iNOS	GGAGAAAACCCCAGGTGCTATT	TCGATGGAGTCACATGCAGC
MCP-1	GATCCCAATGAGTCGGCTGG	ACAGAAGTGCTTGAGGTGGTT
IP-10	GAATCCGGAATCTGAGGCCA	ACGGAGCTCTTTTTGACCTTCT
IL-15	TACTGCAATGAACTGCTTTCTCC	GCTGTTTGCAAGGTAGAGCAC
RIPK1	GACCGAGTTCACAACCACCA	TGTTAGCGAAGACGGCTTGA
RIPK3	TATGGCTCAATGGTGCGTCA	AGTCCCACTCGAGGTTCTCA
BAD	GAATGAGCGATGAATTTGAGGG	CCTTTCCCCAAATTTCGATCC
BID	TGGACTCTGAGGTCAGCAATG	TTCGGAGAAAGCCGAACACC
Caspase 3	AGAGCTGGACTGCGGTATTG	TCGGCCTCCACTGGTATCTT
Caspase 9	GAGGATATTCAGCGGGCAGG	GCAGGAGATGAAGCGAGGAA

### Gene expression in pancreatic islets and ASCs after co-culture or treatment with Sq-GusNPs and inflammatory cytokines

To determine how Sq-GusNPs, ASCs, and inflammatory cytokines affect gene expression in pancreatic islets, 40 handpicked islets were cultured alone or cocultured with ASCs (50.000 ASCs/mL) without contact in presence or absence of Sq-GusNPs at a concentration of 22.31 µg/mL and/or a blend of proinflammatory cytokines (IFN-γ 2000 U/mL, IL-1β 150 U/mL, and TNF-α 2000 U/mL) in 24 well Transwell plates in a total volume of 1 mL culture medium. After 24 hours incubation, the expression of different genes associated with cytokine secretion was determined by qRT-PCR. Islets and ASCs without treatment were used as control group.

### Statistics

The experiments were performed 5 times and statistical analysis was carried out in GraphPad Prism, Version 8.2.0 (GraphPad Software Inc., USA). Normal distribution of data was confirmed using the D’Agostino-Pearson omnibus (K2) test. Where indicated data transformation using the function Y = Log(Y) was applied for analysis. Comparisons were done using one- or two-way ANOVA with Dunnett’s post hoc test. A p-value <0.05 was considered statistically significant. Results are expressed as mean ± standard error of the mean (SEM).

## Results

Recently, we developed Sq-GusNPs, a technology for reducing inflammatory responses in the immediate period post-transplantation that involves nanoencapsulation and temporary, controlled release of the anti-inflammatory agent gusperimus in the vicinity of the graft.^20^,^21^ Here, we investigate whether immunosuppression with Sq-GusNPs in the presence or absence of ASCs can contribute to reduced release of cytokines in the islets or reduce initiation of cell-death processes.

For this study, we co-cultured islets with ASCs and/or Sq-GusNPs under IFN-γ, IL-1β, and TNF-α induced inflammatory stress. This was done with both human and rat islets to determine species specificity. It is well known that this inflammatory stress does not immediately lead to impaired viability or reduced insulin secretion in the first 24 hours of exposure. Also, here we did not find any effects on viability or the glucose-induced insulin release (Figure S1 and S2 of the supplementary information). It has been reported that this exposure to IFN-γ, IL-1β, and TNF-α does influence the islets cytokine and chemokine secretion and other factors associated with an inflammatory response.^27^,^28^ Therefore, we determined here the impact of IFN-γ, IL-1β, and TNF-α in the presence and absence of ASCs and/or Sq-GusNPs on gene expression for both islets and ASCs. We focused on the expression of the nuclear factor kappa-light-chain-enhancer of activated B cells (NF-κB) and genes associated with the activation of the NF-κB route, namely, the oxidative stress indicator inducible nitric oxide synthase (iNOS), and relevant cytokines, chemokines, and cell-death genes.

### Impact of Sq-GusNPs and co-culture on NF-κB gene expression in islets and ASCs

**Figure 1.**
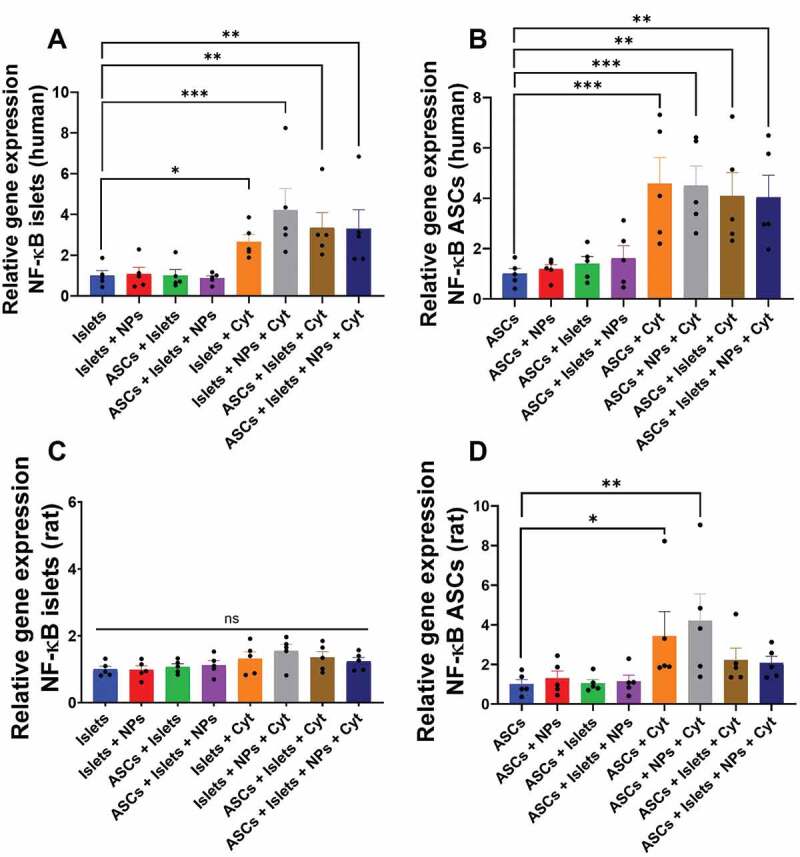
NF-κB gene expression in human and rat islets and ASCs. Human or rat pancreatic islets were co-cultured in the presence and absence of IFN-γ, IL-1β, and TNF-α with ASCs, Sq-GusNPs, or a combination of both. A) NF-κB expression in human islets. B) NF-κB expression in human ASCs. C) NF-κB expression in rat islets. D) NF-κB expression in rat ASCs. Comparisons were made using one-way ANOVA with Dunnet’s multiple comparison test after data transformation using the function Y = Log(Y). Islets (Islets without treatment); Islets + NPs (Islets treated with Sq-GusNPs); ASCs + Islets (Adipose-derived stem cells co-cultured with pancreatic islets); ASCs + Islets + NPs (Adipose-derived stem cells co-cultured with islets and treated with Sq-GusNPs); Islets + Cyt (Islets stimulated with the cytokine mixture); Islets + NPs + Cyt (Islets treated with Sq-GusNPs and stimulated with the cytokine mixture); ASCs + Islets + Cyt (Adipose-derived stem cells co-cultured with islets and stimulated with cytokine cocktail); ASCs + Islets + NPs + Cyt (Adipose-derived stem cells co-cultured with islets, treated with Sq-GusNPs, and stimulated with the cytokine mixture). Data represent mean values ± SEM of five independent experiments. p < .0009 (***); p ≤ .005 (**); p < .05 (*).

We first studied how IFN-γ, IL-1β, and TNF-α exposure in presence or absence of Sq-GusNPs and/or co-culture influenced the expression of the NF-κB gene in human and rat islets and ASCs. Human islets and ASCs showed no upregulation of NF-κB under basal conditions but exposure to the cytokine mixture did upregulate its expression in both human islets and ASCs (Figure 1A and 1B). The cytokine mixture increased the expression of the NF-κB gene by 2.9-fold (p = .0106) in human islets compared to untreated, control islets (Figure 1A). In human ASCs the cytokine-induced enhancement of NF-κB was 4.5-fold (p = .0006) compared to untreated, control ASCs (Figure 1B). No attenuation of NF-κB expression was observed for cytokine-exposed human islets or ASCs when co-cultured with either Sq-GusNPs or ASCs or a combination thereof (Table S1 of the supplementary information).

Rat islets showed no upregulation of NF-κB gene expression neither under basal nor under cytokine exposed conditions (Figure 1C). This was different in ASCs, as rat ASCs showed an increase in the expression of NF-κB of 3.2-fold (p < .05) after IFN-γ, IL-1β, and TNF-α exposure (Figure 1D). Treatment of cytokine exposed rat ASCs with Sq-GusNPs did not reduce or inhibit NF-κB. However, co-culture of ASCs with rat islets and addition of Sq-GusNPs to the stimulated co-culture avoided the upregulation of the NF-κB gene (Table S2 of the supplementary information).

### Impact of Sq-GusNPs and co-culture on iNOS gene expression in islets and ASCs

As inflammatory stress induces nitric oxide (NO) production by islets,^29^ we studied how exposure to the cytokine mixture contributes to iNOS expression in human and rat islets and ASCs. iNOS is a gene that encodes the inducible nitric oxide synthase enzyme responsible for the production of NO from L-arginine in presence of NADPH and oxygen.^30^

**Figure 2.**
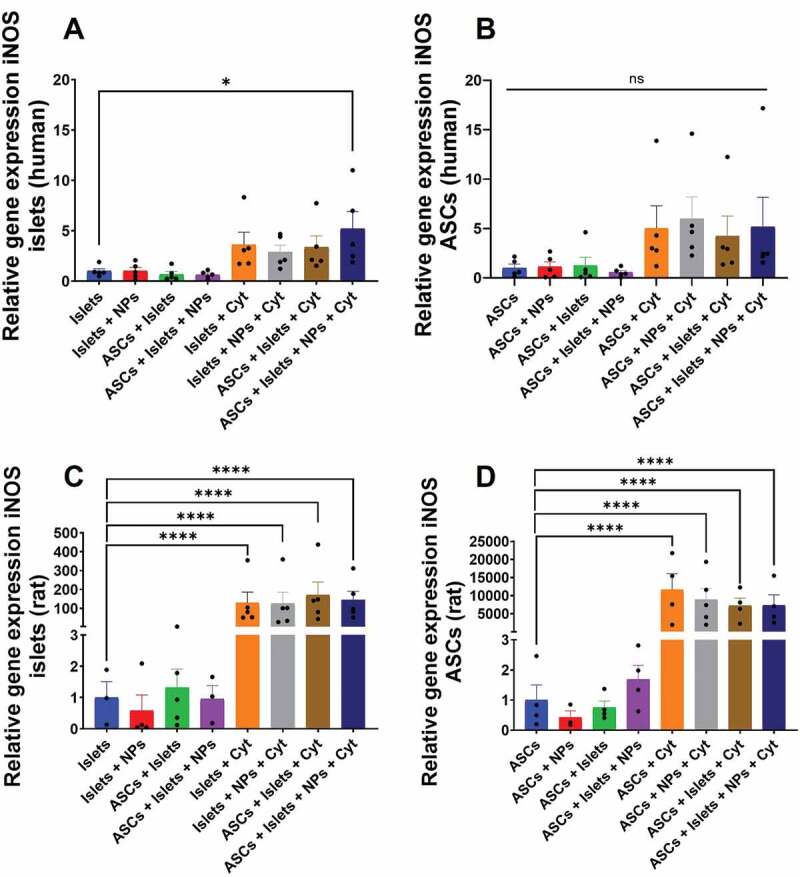
iNOS gene expression in human and rat islets and ASCs. Human or rat pancreatic islets were co-cultured in the presence and absence of IFN-γ, IL-1β, and TNF-α with ASCs, Sq-GusNPs, or a combination of both. A) iNOS expression in human islets. B) iNOS expression in human ASCs. C) iNOS expression in rat islets. D) iNOS expression in rat ASCs. Comparisons were made using one-way ANOVA with Dunnet’s multiple comparison test after data transformation using the function Y = Log(Y). Islets (Islets without treatment); Islets + NPs (Islets treated with Sq-GusNPs); ASCs + Islets (Adipose-derived stem cells co-cultured with pancreatic islets); ASCs + Islets + NPs (Adipose-derived stem cells co-cultured with islets and treated with Sq-GusNPs); Islets + Cyt (Islets stimulated with the cytokine mixture); Islets + NPs + Cyt (Islets treated with Sq-GusNPs and stimulated with the cytokine mixture); ASCs + Islets + Cyt (Adipose-derived stem cells co-cultured with islets and stimulated with cytokine cocktail); ASCs + Islets + NPs + Cyt (Adipose-derived stem cells co-cultured with islets, treated with Sq-GusNPs, and stimulated with the cytokine mixture). Data represent mean values ± SEM of five independent experiments. p < .0001 (****); p < .05 (*); ns (non-significant statistical difference).

In human islets, increase in the expression of the iNOS gene was only observed when cytokine exposed islets were co-cultured with ASCs in presence of Sq-GusNPs which enhanced iNOS expression 4.6-fold (p = .0107) (Figure 2A, Table S3 of the supplementary information). Human ASCs showed no increase in gene expression for iNOS neither unstimulated nor when exposed to cytokines (Figure 2B).

Rat cells showed to be more sensitive for cytokine-induced iNOS expression. Rat islets showed a profound increase of 152.7-fold in iNOS expression after cytokine exposure (Figure 2C). Rat ASCs showed a rather high increase in iNOS expression compared to rat islets by 12455.1-fold (Figure 2D) after cytokine exposure. Co-culture and/or treatment with Sq-GusNPs did not reduce or increase the expression of this gene under cytokine-stimulated conditions significantly (Table S4 of the supplementary information).

### Impact of Sq-GusNPs and co-culture on inflammatory cytokines/chemokines-associated gene expression in islets and ASCs

Next, we studied how IFN-γ, IL-1β, and TNF-α exposure in presence and absence of Sq-GusNPs and/or ASCs influence the expression of specific genes associated with the activation of the NF-κB route and support the expression of key cytokines and chemokines in human and rat islets and ASCs under inflammatory or basal conditions.

#### Inflammatory cytokines-associated gene expression

**Figure 3.**
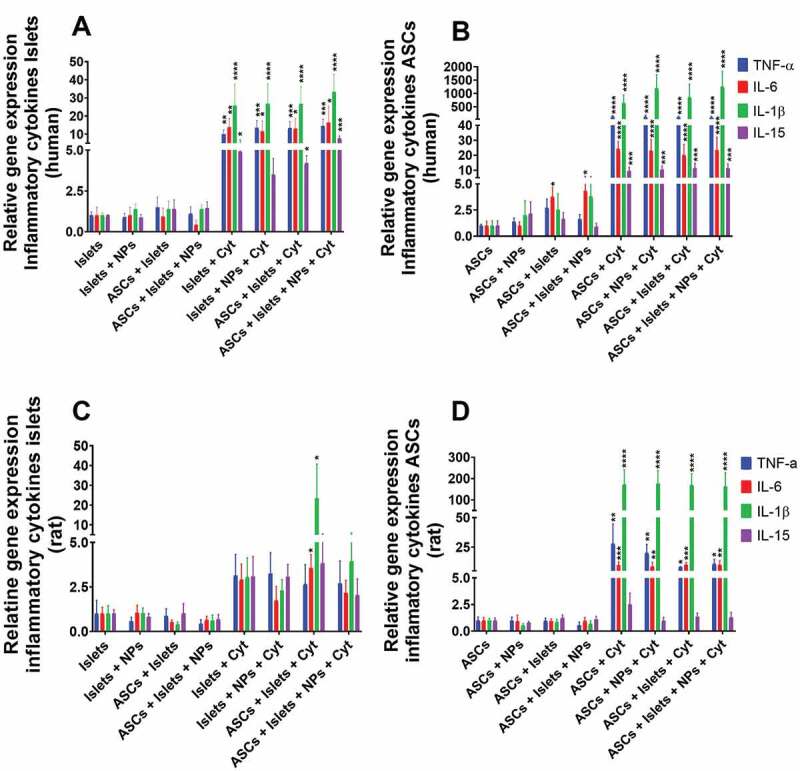
Gene expression associated with secretion of proinflammatory cytokines for human and rat islets and ASCs. Human or rat pancreatic islets were co-cultured in the presence and absence of IFN-γ, IL-1β, and TNF-α with ASCs, Sq-GusNPs, or a combination of both. A) Inflammatory gene expression in human islets. B) Inflammatory gene expression in human ASCs. C) Inflammatory gene expression in rat islets. D) Inflammatory gene expression in rat ASCs. Comparisons were made using one-way ANOVA with Dunnet’s multiple comparison test after data transformation using the function Y = Log(Y). Islets (Islets without treatment); Islets + NPs (Islets treated with Sq-GusNPs); ASCs + Islets (Adipose-derived stem cells co-cultured with pancreatic islets); ASCs + Islets + NPs (Adipose-derived stem cells co-cultured with islets and treated with Sq-GusNPs); Islets + Cyt (Islets stimulated with the cytokine mixture); Islets + NPs + Cyt (Islets treated with Sq-GusNPs and stimulated with the cytokine mixture); ASCs + Islets + Cyt (Adipose-derived stem cells co-cultured with islets and stimulated with cytokine cocktail); ASCs + Islets + NPs + Cyt (Adipose-derived stem cells co-cultured with islets, treated with Sq-GusNPs, and stimulated with the cytokine mixture). Data represent mean values ± SEM of five independent experiments. p < .0001 (****); p < .0009 (***); p < .005 (**); p < .05 (*).

Here, we studied the expression of the genes associated with secretion of the cytokines tumor necrosis factor-alpha (TNF-α), interleukin 6 (IL-6), interleukin 1 beta (IL-1β), and interleukin 15 (IL-15). In absence of an inflammatory stimulus, we did not observe increase in gene expression in human nor in rat islets in presence or absence of either ASCs and/or Sq-GusNPs (Figure 3A and 3C). Cytokine exposure of human islets enhanced the expression of all the evaluated genes (Figure 3A). TNF-α was increased by 8.8-fold (p = .0011), IL-6 was increased by 26.6-fold (p = .008), IL-1β was increased by 20.4-fold (p < .0001), and IL-15 was increased by 3.7-fold (p = .0196). Treatment of the stimulated islets with Sq-GusNPs inhibited the increase of IL-15 but did not inhibit the increased expression of TNF-α, IL-6, and IL-1β. Co-incubation with ASCs or co-incubation and treatment with Sq-GusNPs did not reduce the expression of the evaluated genes after cytokine stimulation (Table S5 of the supplementary information).

We also determined the expression of these genes in ASCs and how islets might influence gene expression in ASCs by crosstalk. As shown in Figure 3B, ASCs had an enhanced expression of the IL-6 gene when exposed to human islets. Human islets enhanced expression of IL-6 by 5.2-fold (p = .0292) in ASCs in absence of the proinflammatory cytokines. The increased expression was not inhibited for the treatment with Sq-GusNPs and was maintained 5.2-fold (p = .0293) higher than in the control group (untreated ASCs). Under cytokine stimulation, ASCs increased expression of TNF-α, IL-6, IL-1β, and IL-15 by 105.2-fold (p < .0001), 36.3-fold (p < .0001), 389.0-fold (p < .0001), and 11.8-fold (p = .0007) respectively. No significative reduction or inhibition in gene expression for cytokine-stimulated ASCs was observed when these cells were co-cultured with islets and/or treated with Sq-GusNPs (Table S6 of the supplementary information).

Rat islets showed a different expression profile than human islets with fewer expression of proinflammatory cytokine genes. No increase in the expression of these genes was seen when islets were cultured with the cytokine mixture with or without Sq-GusNPs (Figure 3C). When islets were co-cultured with ASCs IL-6 and IL-1β expression was increased by 4.4- (p = .0274), and 9.9-times (p = .0261) respectively in presence of cytokines. Addition of Sq-GusNPs to the stimulated co-culture inhibited the expression of both cytokines (Table S7 of the supplementary information).

Also, with the rat experiments, we studied the impact of cytokine exposure, co-culture, and Sq-GusNPs treatment in rat ASCs gene expression (Figure 3D). TNF-α, IL-6, and IL-1β expression was increased by 17.0-fold (p = .0034), 12.5-fold (p = .0009), and 118.6-fold (p < .0001) respectively. This increased expression was not reduced significantly by Sq-GusNPs exposure, co-culture with rat islets, or a combination thereof. Noticeably, the rat ASCs were strong producers of IL-1β and unlike human ASCs, rat ASCs did not increase the expression of IL-15 after exposure to the cytokine mixture (Table S8 of the supplementary information).

#### Inflammatory chemokines-associated gene expression

**Figure 4.**
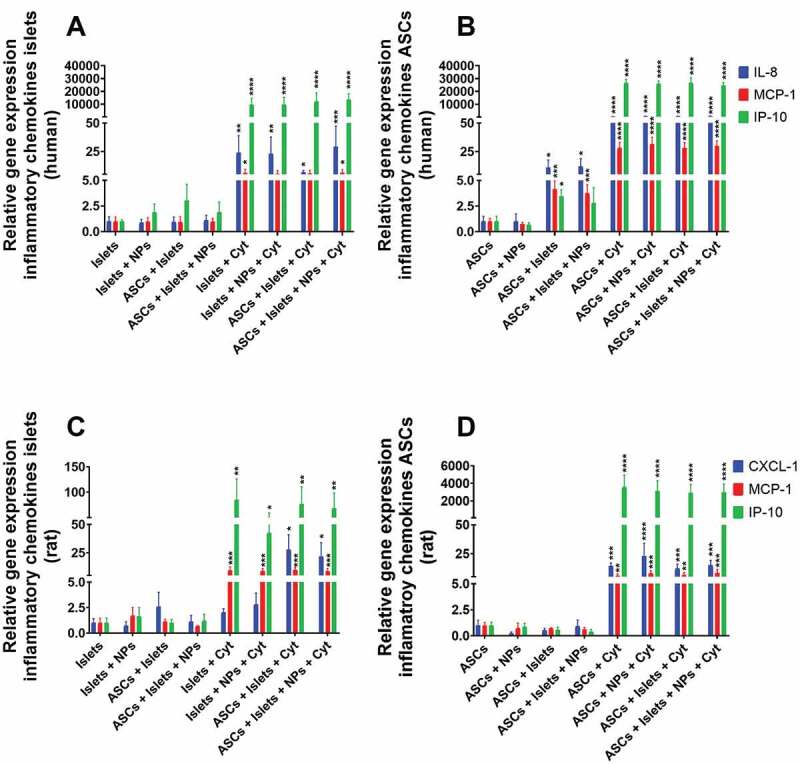
Gene expression associated with secretion of proinflammatory chemokines for human and rat islets and ASCs. Human or rat pancreatic islets were co-cultured in the presence and absence of IFN-γ, IL-1β, and TNF-α with ASCs, Sq-GusNPs, or a combination of both. A) Chemokine expression in human islets. B) Chemokine expression in human ASCs. C) Chemokine expression in rat islets. D) Chemokine expression in rat ASCs. Comparisons were made using one-way ANOVA with Dunnet’s multiple comparison test after data transformation using the function Y = Log(Y). Islets (Islets without treatment); Islets + NPs (Islets treated with Sq-GusNPs); ASCs + Islets (Adipose-derived stem cells co-cultured with pancreatic islets); ASCs + Islets + NPs (Adipose-derived stem cells co-cultured with islets and treated with Sq-GusNPs); Islets + Cyt (Islets stimulated with the cytokine mixture); Islets + NPs + Cyt (Islets treated with Sq-GusNPs and stimulated with the cytokine mixture); ASCs + Islets + Cyt (Adipose-derived stem cells co-cultured with islets and stimulated with cytokine cocktail); ASCs + Islets + NPs + Cyt (Adipose-derived stem cells co-cultured with islets, treated with Sq-GusNPs, and stimulated with the cytokine mixture). Data represent mean values ± SEM of five independent experiments. p < .0001 (****); p < .0009 (***); p < .005 (**); p < .05 (*).

We also studied some key genes associated with secretion of inflammatory chemokines whose production is induced by inflammatory cytokines via the NF-κB route, namely, interleukin 8 (IL-8) in humans or its equivalent cytokine in rat chemokine (C-X-C motif) ligand 1 (CXCL-1), monocyte chemoattractant protein-1 (MCP-1), and interferon-gamma inducible protein-10 (IP-10). In absence of cytokine exposure, no increase in the expression of the evaluated chemokines was observed neither in human or rat islets even when they were co-cultured with ASCs irrespective of the presence of Sq-GusNPs (Figure 4A and 4C).

When human islets were exposed to the cytokine mixture all the studied chemokine genes were upregulated (Figure 4A). The expression was increased by 19.5-fold for IL-8 (p = .0015), 5.0-fold for MCP-1 (p = .0403), and an extreme enhancement of 6456.5-fold (p < .0001) was observed for IP-10. Sq-GusNPs or co-culture with human ASCs inhibited the expression of MCP-1 and maintained an increased expression of IL-8 and IP-10 in cytokine-stimulated conditions (Table S9 of the supplementary information). For human islets co-cultured with ASCs and treated with Sq-GusNPs no inhibition in gene expression under inflammatory stimulus was observed and all the evaluated chemokines were upregulated. For IL-8, MCP-1, and IP-10 the expression was increased by 23.6-fold (p = .0007), 5.9-fold (p = .0192), and 10715.2-fold (p < .0001) respectively.

Under basal conditions human islets induced the expression of IL-8, MCP-1, and IP-10 by 8.4-fold (p = .0325), 4.6-fold (p = .0008), and 5.7-fold (p = .0184) respectively in human ASCs (Figure 4B). Addition of Sq-GusNPs to the co-culture without cytokine stimulation did not inhibit IL-8 and MCP-1 expression but did inhibit the expression of IP-10 in ASCs (Table S10 of the supplementary information). Human ASCs upregulated gene expression for all the evaluated genes when they were exposed to the cytokine mixture. IL-8 was increased by 554.6-fold (p < .0001), MCP-1 was increased by 31.9-fold (p < .0001), and as for human islets, IP-10 expression was increased to a higher extent by 46131.8-fold (p < .0001). When stimulated ASCs were treated with Sq-GusNPs, co-cultured with human islets, or co-cultured and treated with Sq-GusNPs no inhibition in gene expression was observed (Table S10 of the supplementary information).

Rat islets showed an increased expression of two of the evaluated genes with a higher increase of IP-10 when they were stimulated with the cytokine mixture (Figure 4C). MCP-1 was increased by 10.7-fold (p = .0003) and IP-10 was increased by 58.3-fold (p = 0.0011). This expression was not reduced significantly when rat islets were co-cultured with ASCs, treated with Sq-GusNPs, or co-cultured and treated with Sq-GusNPs under inflammatory stimulus (Table S11 of the supplementary information). Interestingly, when stimulated rat islets were co-cultured with ASCs an increase in the expression of CXCL-1 by 22.2-fold (p = 0.0124) was observed. Addition of Sq-GusNPs to the stimulated co-culture did not inhibit the increased expression of CXCL-1.

In rat ASCs, the expression of CXCL-1, MCP-1, and IP-10 was also increased under inflammatory conditions with a further elevated expression for IP-10 in comparison to rat islets (Figure 4D). The CXCL-1 expression was increased by 28.5-fold (p = 0.0001), MCP-1 was increased by 6.2-fold (p = 0.003), and IP-10 was increased by 2517.7-fold (p < 0.0001) when ASCs were exposed to the cytokine mixture. When rat ASCs were treated with Sq-GusNPs and/or co-cultured with islets in presence of the cytokine mixture no reduction or inhibition in the expression of any of these genes was observed (Table S12 of the supplementary information).

### Impact of Sq-GusNPs and co-culture on anti-inflammatory cytokines-associated gene expression in islets and ASCs

**Figure 5.**
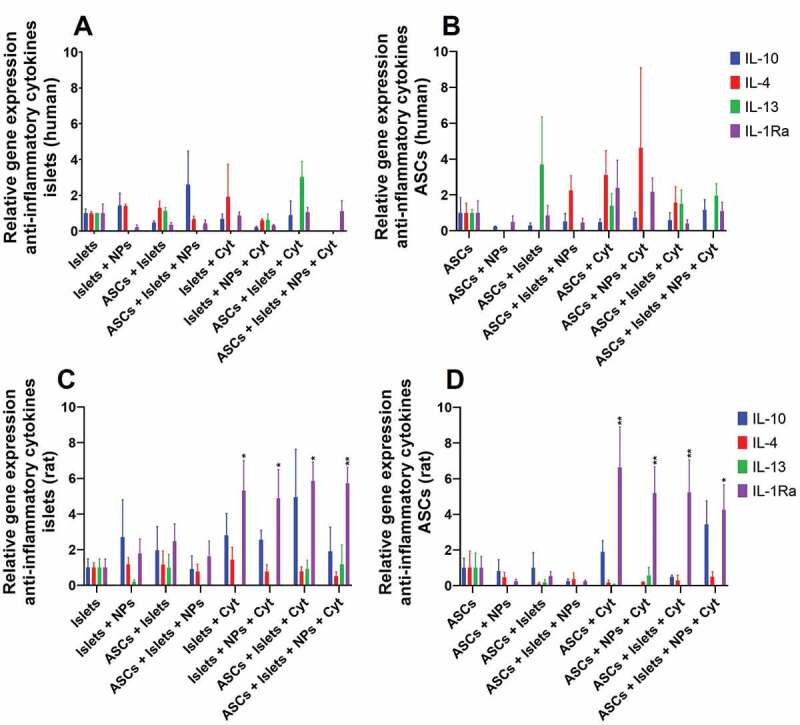
Regulatory cytokine expression in human and rat islets and ASCs. Human or rat pancreatic islets were co-cultured in the presence and absence of IFN-γ, IL-1β, and TNF-α with ASCs, Sq-GusNPs, or a combination of both. A) Regulatory cytokine expression in human islets. B) Regulatory cytokine expression in human ASCs. C) Regulatory cytokine expression in rat islets. D) Regulatory cytokine expression in rat ASCs. Comparisons were made using one-way ANOVA with Dunnet’s multiple comparison test after data transformation using the function Y = Log(Y). Islets (Islets without treatment); Islets + NPs (Islets treated with Sq-GusNPs); ASCs + Islets (Adipose-derived stem cells co-cultured with pancreatic islets); ASCs + Islets + NPs (Adipose-derived stem cells co-cultured with islets and treated with Sq-GusNPs); Islets + Cyt (Islets stimulated with the cytokine mixture); Islets + NPs + Cyt (Islets treated with Sq-GusNPs and stimulated with the cytokine mixture); ASCs + Islets + Cyt (Adipose-derived stem cells co-cultured with islets and stimulated with cytokine cocktail); ASCs + Islets + NPs + Cyt (Adipose-derived stem cells co-cultured with islets, treated with Sq-GusNPs, and stimulated with the cytokine mixture). Data represent mean values ± SEM of five independent experiments. p < .009 (**); p < .05 (*).

In addition to the pro-inflammatory genes, we also studied the expression of the regulatory cytokines interleukin 10 (IL-10), interleukin 4 (IL-4), interleukin 13 (IL-13), and interleukin 1 receptor antagonist (IL-1Ra) in human and rat islets and ASCs. In human islets, we did not observe any statistically significant effect on IL-10, IL-4, IL-13, and IL-1Ra in the absence or presence of IFN-γ, IL-1β, and TNF-α (Figure 5A). Also, co-culture with ASCs and/or treatment with Sq-GusNPs did not influence expression profiles. The same applied to human ASCs (Figure 5B).

Results were different for the rat experiments. Expression of IL-1Ra in rat islets was increased by 5.7-fold (p = 0.0253) when stimulated with IFN-γ, IL-1β, and TNF-α (Figure 5C). Treatment of the stimulated islets with Sq-GusNPs and/or co-culture with ASCs did not inhibit the expression of IL-1Ra. The expression when stimulated islets were treated with Sq-GusNPs was increased 5.3-fold (p = 0.0354) and co-culture with ASCs or a combination of the two increased the expression by 7.6-fold (p < 0.05) compared to untreated islets.

In the rat ASCs, a similar behavior was observed. The cytokine challenge increased IL-1Ra expression 11.6-fold (p = 0.004). Treatment of the stimulated ASCs with Sq-GusNPs or co-culture with islets increased the expression to a lower extent by 9.7- (p = 0.0083), and 9.8-times (p = 0.0079) respectively. A lower increase of 7.9-times (p = 0.0187) in the expression of this gene under cytokine stimulation was observed when ASCs were co-cultured with islets and treated with Sq-GusNPs (Figure 5D).

### Impact of Sq-GusNPs and co-culture on cell death-associated gene expression in islets and ASCs

Finally, we evaluated the expression of six genes associated with cell death processes, two genes associated with necroptosis, receptor-interacting serine/threonine-protein kinase 1 (RIPK1) and receptor-interacting serine/threonine-protein kinase 3 (RIPK3), and four genes associated with cell death via apoptosis, namely, BCL2 associated agonist of cell death (BAD), BH3 interacting-domain death agonist (BID), cysteine-aspartic acid protease 3 (Caspase 3), and cysteine-aspartic acid protease 9 (Caspase 9).

**Figure 6.**
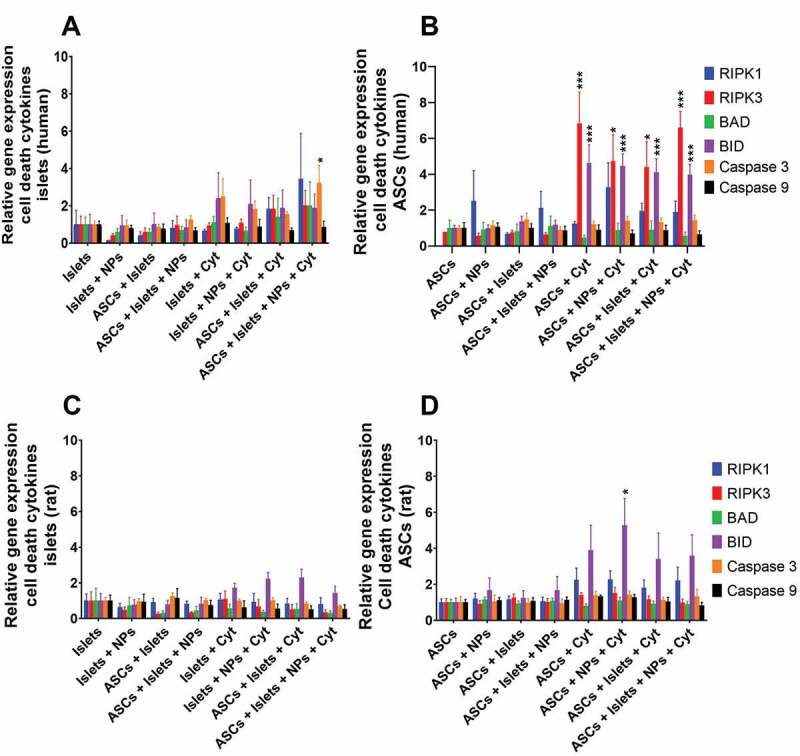
Cell-death associated gene expression in human and rat islets and ASCs. Human or rat pancreatic islets were co-cultured in the presence and absence of IFN-γ, IL-1β, and TNF-α with ASCs, Sq-GusNPs, or a combination of both. A) Gene expression in human islets. B) Gene expression in human ASCs. C) Gene expression in rat islets. D) Gene expression in rat ASCs. Comparisons were made using one-way ANOVA with Dunnet’s multiple comparison test after data transformation using the function Y = Log(Y). Islets (Islets without treatment); Islets + NPs (Islets treated with Sq-GusNPs); ASCs + Islets (Adipose-derived stem cells co-cultured with pancreatic islets); ASCs + Islets + NPs (Adipose-derived stem cells co-cultured with islets and treated with Sq-GusNPs); Islets + Cyt (Islets stimulated with the cytokine mixture); Islets + NPs + Cyt (Islets treated with Sq-GusNPs and stimulated with the cytokine mixture); ASCs + Islets + Cyt (Adipose-derived stem cells co-cultured with islets and stimulated with cytokine cocktail); ASCs + Islets + NPs + Cyt (Adipose-derived stem cells co-cultured with islets, treated with Sq-GusNPs, and stimulated with the cytokine mixture). Data represent mean values ± SEM of five independent experiments. p ≤ .0009 (***); p < .05 (*).

In human islets, we observed no upregulation of the evaluated genes when exposed to IFN-γ, IL-1β, and TNF-α. Only when human islets were co-cultured with ASCs, treated with Sq-GusNPs, and stimulated with cytokines a significant increase of 2.9 times (p < 0.05) of caspase 3 expression was observed (Figure 6A). Human ASCs showed an increased expression of the necroptotic gene RIPK3 and the apoptotic gene BID after stimulation with the cytokine mixture by 6.3-fold (p = 0.0008) and 4.2-fold (p = 0.0004), respectively (Figure 6B). After treatment of the stimulated ASCs with Sq-GusNPs, co-culture with human islets, or co-culture and treatment with Sq-GusNPs no decrease in the upregulated genes was observed (Table S13 of the supplementary information).

In rat islets, no upregulation of any of the evaluated genes was observed. Not under basal or when exposed to the cytokine mixture. The treatment with Sq-GusNPs, co-culture with ASCs, or a combination thereof did not change this (Figure 6C). Rat ASCs only showed upregulation of the pro-apoptotic gene BID when ASCs were stimulated with cytokines and treated with Sq-GusNPs. This induced 5.3 times (p = 0.0131) increase in BID expression (Figure 6D).

## Discussion

Here we studied, to the best of our knowledge, for the first time how pancreatic islets and ASCs mutually influence each other in the presence or absence of an immunosuppressive agent and under inflammatory or basal conditions. To this end, we studied in islets and ASCs genes related to proinflammatory and regulatory responses as well as genes involved in different cell-death processes. We choose to focus on the first 24 hours of exposure to cytokines as this is the period in which insulin secretion is not yet impaired but cellular molecular processes are dysregulated.^27^,^31^ We demonstrated that in the first 24 hours viability or function was not impaired in human or rat islets. Also, the viability of ASCs was not affected. This allowed us to study in islets the effects of co-culture with ASCs and/or treatment with Sq-GusNPs in a period in which irreversible damage to ß-cells is not yet complete.

Our data show that in human or rat islets in absence of cytokine exposure the coculture with ASCs, treatment with Sq-GusNPs, or a combination of both did not enhance the expression of inflammatory cytokines or chemokines. This indicates that Sq-GusNPs or ASCs have no immunogenic influence on pancreatic islets. However, we found that in human ASCs the expression of IL-6, IL-8, MCP-1, and IP-10 was enhanced by human islets in absence of cytokine stimulation. The enhanced expression of these cytokines in human ASCs should not be considered as an observation that negatively impacts graft survival. We consider the expression of IL-6, IL-8, and MCP-1 induced by human islets in human ASCs as beneficial for engraftment as these chemokines have been proposed to be not only inflammatory but also to be proangiogenic,^32–34^ anti-apoptotic, proliferative, and having tissue repair effects through paracrine mechanisms.^35–37^,^14^ This observation suggests that co-incubation of human islets with ASCs in the pre-transplantation period might precondition human ASCs to support engraftment.

The enhanced expression of cytokines in human ASCs induced by human islets was only minorly suppressed by the immunosuppressive agent Sq-GusNPs. Only IP-10 was inhibited. This, however, can also be considered to be beneficial as IP-10 is a potent inhibitor of angiogenesis.^34^ As IL-6, IL-8, and MCP-1 expression was not reduced in human ASCs by Sq-GusNPs we conclude that application of this immunosuppressive regimen does not interfere with processes involved in engraftment of the transplant mediated by human ASCs.

Human cells showed higher activity of NF-κB under inflammatory stress than rat cells indicating a higher sensitivity for inflammatory stress in human cells. This enhanced sensitivity was illustrated by a stronger expression of the studied genes in both human islets as well as in human ASCs. In presence of IFN-γ, IL-1β, and TNF-α, human islets expressed all the studied cytokines and chemokines. In contrast, rat islets did not express any of the studied cytokines, but they did express MCP-1 and IP-10 after cytokine stimulation. Additionally, human ASCs showed a rather high expression of all the evaluated cytokines and chemokines under inflammatory stress than rat ASCs. The observation of higher sensitivity for cytokine stress could explain why human islets compared to rat islets perform worse in different transplantation models, which up to now is usually attributed to differences in islet-quality and lower engraftment of human islets compared to rat islets.^8^

The generation of NO has been identified as one of the main mediators of cytokine-induced β-cell damage.^29^ iNOS gene expression is an indicator of the production of NO by cells.^30^ NO secretion in β-cells has been associated with inhibition of insulin secretion, mitochondrial dysfunction, and DNA damage which can lead to cell death.^28^,^38^ In rat cells, in contrast to human cells, proinflammatory conditions did induce nitrosative stress as was observed in both rat islets and in rat ASCs which both expressed iNOS to a high extend. This might indicate that nitrosative stress is less involved in human islet cell death than in rat islets under inflammatory conditions.^38^,^39^

After 24 hours exposure of human islets to IFN-γ, IL-1β, and TNF-α, Sq-GusNPs inhibited IL-15 and MCP-1. IL-15 is a potent growth factor, chemoattractant, and activator for T cells and natural killer (NK) cells.^34^ MCP-1 induces chemotaxis of monocytes, recruits T cells, and NK cells.^40^ This suggests that Sq-GusNPs can avoid migration and activation of different kinds of cells from the innate and adaptative arms of the immune system and therewith contribute to the prevention of graft rejection by directly suppressing cytokines in human islets under inflammatory stress. Additionally, Sq-GusNPs does also influence immune cells in the vicinity of the graft and have been shown to downregulate the secretion in macrophages of TNF-α, IL-6, and IL-8.^20^ This again demonstrates that immunosuppression by Sq-GusNPs does not negatively but rather beneficially influence processes related to inflammation and engraftment of especially human islets.

Rat islets were less sensitive for the inflammatory cytokines than human islets and showed no upregulation of genes associated with inflammation. However, in contrast to human islets, co-culture of cytokine-exposed rat islets with rat ASCs increased expression of IL-6 and IL-1β in rat islets. Here, Sq-GusNPs exerted an inhibitory effect on the expression of IL-6 and IL-1β in rat islets even under the influence of IL-1β highly expressed by the rat ASCs in co-culture. Again, this illustrates a major difference between human and rat islet-responses to ASCs and Sq-GusNPs. It also shows the protective effect that Sq-GusNPs can have on rat islets in co-culture conditions with ASCs.

Rat islets and ASCs in contrast to human islets and ASCs expressed the regulatory cytokine IL-1Ra. This could be one of the reasons why rat islets were less susceptible to cytokine exposure. IL-1Ra has been shown to neutralize pro-inflammatory cytokines and chemokines such as IL-1β, IL-6, TNF-α, KC, MCP-1, and MIP-1α.^30^,^41^ This finding suggests that rat islets are more tolerant to inflammatory conditions than human islets thanks to autoregulation driven by IL-1Ra secretion. By their part and according to our results, the observation of an increased IL-1Ra expression in rat ASCs suggests that rat ASCs may reinforce anti-inflammatory effects on rat islets through a paracrine effect as has been previously described.^12^

Finally, human and rat islets showed a low tendency or no tendency to express genes related to cell death at 24 hours. This can explain why islet-cell death is not observed at an early stage after inflammatory stimulation. Even human islets, despite their enhanced sensitivity to inflammatory conditions, only showed an increased expression of caspase 3 in co-culture with human ASCs after cytokine exposure and treatment with Sq-GusNPs. This indicates that in principle a combination of immunosuppression with Sq-GusNPs and ASCs may induce cell death by apoptosis in human islets in a caspase 3 dependent pathway under pro-inflammatory conditions. Notably, before apoptosis occurs not only caspase 3 should be activated but also *e.g*. cytochrome c release and caspase 9 should be upregulated to eventually induce cell death.^42^ It remains to be determined whether the combination of co-culture of human islets with human ASCs and treatment with Sq-GusNPs can induce apoptosis in human islets under pro-inflammatory conditions.

Surprisingly, human ASCs showed to be more prone to cell death processes under inflammatory conditions than rat ASCs. Human ASCs increased the expression of the necroptotic gen RIPK3 and the apoptotic gene BID without increasing the expression of the RIPK1 gene. This suggests that inflammatory conditions may induce necroptotic death in human ASCs in a RIPK1 independent mechanism.^43^ This consideration is corroborated by the increased expression of the BID gene in human ASCs, since, even though BID is considered a pro-apoptotic gene,^44^ its expression together with RIPK3 has shown to be involved in complement-dependent cytotoxicity leading to necroptosis in a pathway that depends on both RIPK3 and MLKL.^45^,^46^ For cytokine-stimulated rat ASCs, only the treatment with Sq-GusNPs produced an increased expression of the pro-apoptotic gene BID.^47^ This suggests that Sq-GusNPs may induce cell death via apoptosis in rat ASCs under inflammatory conditions in a BID-dependent pathway.

## Conclusion

The beneficial impact of ASCs on islet cells is not influenced by immunosuppression induced by Sq-GusNPs. Sq-GusNPs treatment did only minorly reduce cytokines and chemokines in human ASCs that are associated with anti-apoptotic, proangiogenic, proliferative, and tissue repair capacity effects. However, there are major differences between human and rat islets. The species-dependent effects were observed after exposure to IFN-γ, IL-1β, and TNF-α as well as when exposed to ASCs and/or treatment with Sq-GusNPs. Exposure to the cytokines had a strong impact on human islets by NF-κB dependent activation but not via nitrosative stress whereas in rat islets the impact was mainly on nitrosative responses and less on NF-κB activation. Rat islets showed higher tolerance to inflammatory conditions which seem to be driven by IL-1Ra secretion by rat islets and rat ASCs. Our data support the use of ASCs and Sq-GusNP to manage inflammatory responses and to support engraftment of human islets pre- and post-transplantation for T1DM treatment. This study is the basis for further studies in which later times, protein expression measurements, and inclusion of additional genes such as cytochrome C and MLKL will be included to corroborate synergistic effects of Sq-GusNPs and ASCs in the functional survival of pancreatic islets.

## Supplementary Material

Supplemental Material

## Data Availability

Data supporting the findings of this study are available within the article, in the supplementary file, and from the corresponding author upon reasonable request.
