# Supplementary material for: Species-dependent impact of immunosuppressive squalene-gusperimus nanoparticles and adipose-derived stem cells on isolated human and rat pancreatic islets
Source: Islets. 2022 Jul 15;14(1):164–83. doi: 10.1080/19382014.2022.2100191 (PMC9291694; doi:10.1080/19382014.2022.2100191)
Supplement: Supplemental Material [file KISL_A_2100191_SM9682.docx]

**Supplementary Information**

**Species-dependent impact of immunosuppressive squalene-gusperimus nanoparticles and adipose-derived stem cells on isolated human and rat pancreatic islets**

**
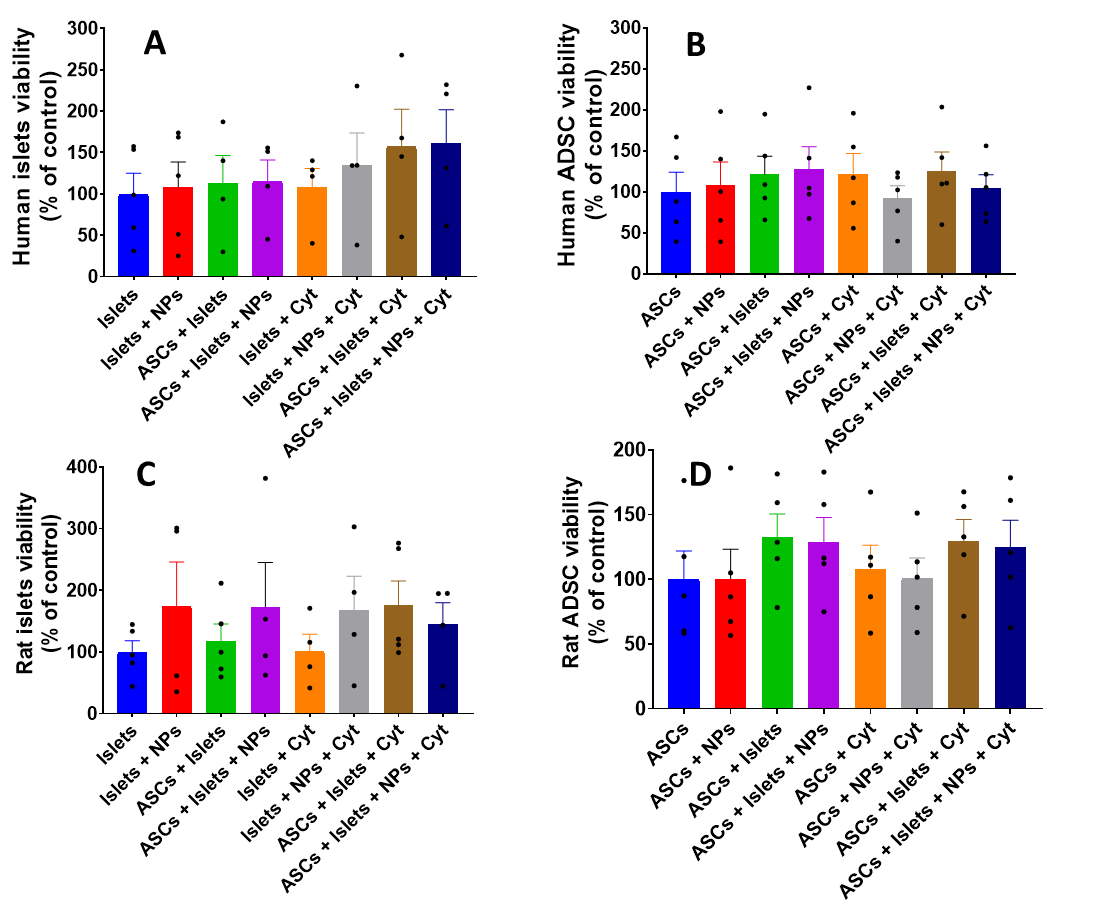
**

**Figure S1:** Viability for human and rat pancreatic islets and ASCs. Human (A and B) or rat (C and D) pancreatic islets and ASCs were co-cultured or cultured alone in presence or absence of Sq-GusNPs or a cytokine cocktail for 24 hours. No differences on viability were found neither for human nor rat cells irrespective of the treatment. Comparisons were made using one-way ANOVA with Dunnet’s multiple comparison test. Islets (Islets without treatment); Islets + NPs (Islets treated with Sq-GusNPs); ASCs + Islets (Adipose-derived stem cells co-cultured with pancreatic islets); ASCs + Islets + NPs (Adipose-derived stem cells co-cultured with islets and treated with Sq-GusNPs); Islets + Cyt (Islets stimulated with the cytokine mixture); Islets + NPs + Cyt (Islets treated with Sq-GusNPs and stimulated with the cytokine mixture); ASCs + Islets + Cyt (Adipose-derived stem cells co-cultured with islets and stimulated with cytokine cocktail); ASCs + Islets + NPs + Cyt (Adipose-derived stem cells co-cultured with islets, treated with Sq-GusNPs, and stimulated with the cytokine mixture). Data represent mean values ± SEM of five independent experiments.

**
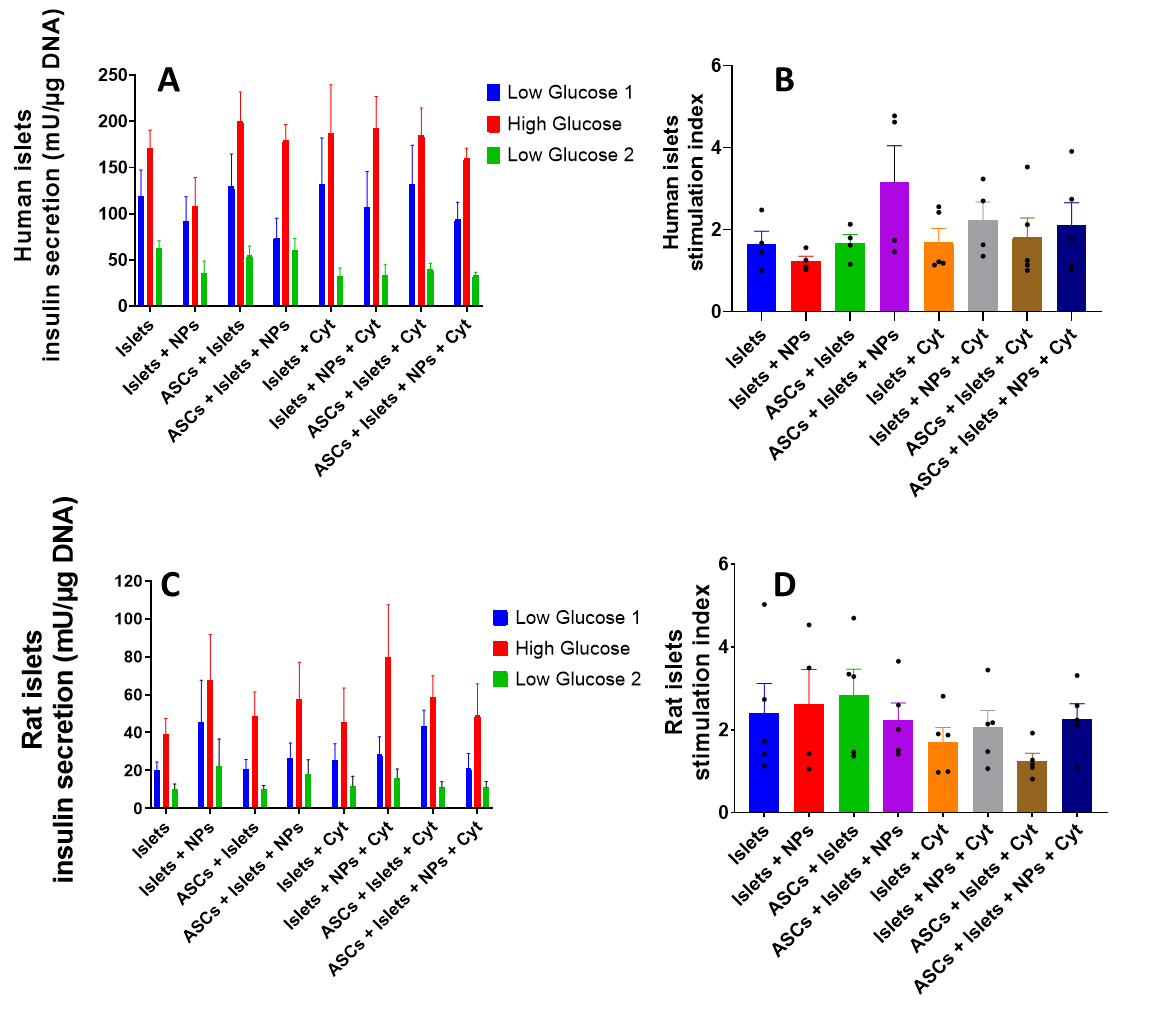
**

**Figure S2.** Evaluation of pancreatic islets function after co-culture with ASCs and treatment with Sq-GusNPs under inflammatory stimulus in the first 24 hours. Human or rat pancreatic islets were co-cultured with ASCs or cultured alone in presence or absence of Sq-GusNPs or a cytokine cocktail for 24 hours. A) GSIS test for human islets after stimulation with low- and high-glucose solution. Comparisons were made using two-way ANOVA with Dunnet’s multiple comparison test after data transformation using the function Y = Log(Y). B) Stimulation index for human islets. Comparisons were made using one-way ANOVA with Dunnet’s multiple comparison test. C) GSIS test for rat islets after stimulation with low- and high-glucose solution. Comparisons were made using two-way ANOVA with Dunnet’s multiple comparison test after data transformation using the function Y = Log(Y). D) Stimulation index for rat islets. Comparisons were made using one-way ANOVA with Dunnet’s multiple comparison test. No impairment of islet function or differences in insulin secretion were found neither for human nor rat islets irrespective of the treatment in the evaluated frame of time. Islets (Islets without treatment); Islets + NPs (Islets treated with Sq-GusNPs); ASCs + Islets (Adipose-derived stem cells co-cultured with pancreatic islets); ASCs + Islets + NPs (Adipose-derived stem cells co-cultured with islets and treated with Sq-GusNPs); Islets + Cyt (Islets stimulated with the cytokine mixture); Islets + NPs + Cyt (Islets treated with Sq-GusNPs and stimulated with the cytokine mixture); ASCs + Islets + Cyt (Adipose-derived stem cells co-cultured with islets and stimulated with cytokine cocktail); ASCs + Islets + NPs + Cyt (Adipose-derived stem cells co-cultured with islets, treated with Sq-GusNPs, and stimulated with the cytokine mixture). Data represent mean values ± SEM of five independent experiments.

**Table S1.** Mean difference for NF-κB gene expression relative to untreated human islets or ASCs.

| ***Comparison*** | ***NF-*κ*B (human islets)*** |  | ***Comparison*** | ***NF-*κ*B (human ASCs)*** |
| --- | --- | --- | --- | --- |
|  |  |  |  |  |
| Islets vs. Islets + NPs | 1.01949 |  | ASCs vs. ASCs + NPs | 1.23740 |
| Islets vs. ASCs + Islets | 1.02773 |  | ASCs vs. ASCs + Islets | 1.40249 |
| Islets vs. ASCs + Islets + NPs | 1.07922 |  | ASCs vs. ASCs + Islets + NPs | 1.41906 |
| Islets vs. Islets + Cyt | 2.86616**•** |  | ASCs vs. ASCs + Cyt | 4.54255**•** |
| Islets vs. Islets + NPs + Cyt | 4.22280**•** |  | ASCs vs. ASCs + NPs + Cyt | 4.69353**•** |
| Islets vs. ASCs + Islets + Cyt | 3.45064**•** |  | ASCs vs. ASCs + Islets + Cyt | 4.11718**•** |
| Islets vs. ASCs + Islets + NPs + Cyt | 3.24265**•** |  | ASCs vs. ASCs + Islets + NPs + Cyt | 4.69353**•** |

**•** *Indicates statistically significant difference respect untreated control (p < 0.05)*

**Table S2.** Mean difference for NF-κB gene expression relative to untreated rat islets or ASCs.

| ***Comparison*** | ***NF-*κ*B (rat islets)*** |  | ***Comparison*** | ***NF-*κ*B (rat ASCs)*** |
| --- | --- | --- | --- | --- |
|  |  |  |  |  |
| Islets vs. Islets + NPs | 1.03058 |  | ASCs vs. ASCs + NPs | 1.24408 |
| Islets vs. ASCs + Islets | 1.08156 |  | ASCs vs. ASCs + Islets | 1.13818 |
| Islets vs. ASCs + Islets + NPs | 1.10622 |  | ASCs vs. ASCs + Islets + NPs | 1.13237 |
| Islets vs. Islets + Cyt | 1.27262 |  | ASCs vs. ASCs + Cyt | 3.24938**•** |
| Islets vs. Islets + NPs + Cyt | 1.51182 |  | ASCs vs. ASCs + NPs + Cyt | 3.87525**•** |
| Islets vs. ASCs + Islets + Cyt | 1.32587 |  | ASCs vs. ASCs + Islets + Cyt | 2.28507 |
| Islets vs. ASCs + Islets + NPs + Cyt | 1.23694 |  | ASCs vs. ASCs + Islets + NPs + Cyt | 2.25165 |

**•** *Indicates statistically significant difference respect untreated control (p < 0.05)*

**Table S3.** Mean difference for iNOS gene expression relative to untreated human islets or ASCs.

| ***Comparison*** | ***iNOS (human islets)*** |  | ***Comparison*** | ***iNOS (human ASCs)*** |
| --- | --- | --- | --- | --- |
|  |  |  |  |  |
| Islets vs. Islets + NPs | 1.19969 |  | ASCs vs. ASCs + NPs | 1.28469 |
| Islets vs. ASCs + Islets | 1.83063 |  | ASCs vs. ASCs + Islets | 1.41514 |
| Islets vs. ASCs + Islets + NPs | 1.66418 |  | ASCs vs. ASCs + Islets + NPs | 1.77705 |
| Islets vs. Islets + Cyt | 3.33196 |  | ASCs vs. ASCs + Cyt | 5.34318 |
| Islets vs. Islets + NPs + Cyt | 2.77971 |  | ASCs vs. ASCs + NPs + Cyt | 7.14825 |
| Islets vs. ASCs + Islets + Cyt | 3.09101 |  | ASCs vs. ASCs + Islets + Cyt | 4.45041 |
| Islets vs. ASCs + Islets + NPs + Cyt | 4.63234**•** |  | ASCs vs. ASCs + Islets + NPs + Cyt | 4.81504 |

**•** *Indicates statistically significant difference respect untreated control (p < 0.05)*

**Table S4.** Mean difference for iNOS gene expression relative to untreated rat islets or ASCs.

| ***Comparison*** | ***iNOS (rat islets)*** |  | ***Comparison*** | ***iNOS (rat ASCs)*** |
| --- | --- | --- | --- | --- |
|  |  |  |  |  |
| Islets vs. Islets + NPs | 0.24199 |  | ASCs vs. ASCs + NPs | 1.97606 |
| Islets vs. ASCs + Islets | 1.20526 |  | ASCs vs. ASCs + Islets | 1.00593 |
| Islets vs. ASCs + Islets + NPs | 1.08116 |  | ASCs vs. ASCs + Islets + NPs | 2.18172 |
| Islets vs. Islets + Cyt | 152.75661**•** |  | ASCs vs. ASCs + Cyt | 12445.14612**•** |
| Islets vs. Islets + NPs + Cyt | 128.52867**•** |  | ASCs vs. ASCs + NPs + Cyt | 9931.16048**•** |
| Islets vs. ASCs + Islets + Cyt | 201.83664**•** |  | ASCs vs. ASCs + Islets + Cyt | 9120.10839**•** |
| Islets vs. ASCs + Islets + NPs + Cyt | 187.93168**•** |  | ASCs vs. ASCs + Islets + NPs + Cyt | 9931.16048**•** |

**•** *Indicates statistically significant difference respect untreated control (p < 0.05)*

**Table S5.** Mean difference for inflammatory cytokines-associated gene expression relative to untreated human islets.

|  | ***Gene (human islets)*** | | | |
| --- | --- | --- | --- | --- |
| ***Comparison*** | ***TNF-α*** | ***IL-6*** | ***IL-1β*** | ***IL-15*** |
| Islets vs. Islets + NPs | 1.25406 | 1.02232 | 1.28766 | 1.247671 |
| Islets vs. ASCs + Islets | 1.19479 | 1.42495 | 1.18872 | 1.043903 |
| Islets vs. ASCs + Islets + NPs | 1.01438 | 1.89496 | 1.38580 | 1.276439 |
| Islets vs. Islets + Cyt | 8.78011**•** | 26.60725**•** | 20.41738**•** | 3.743691**•** |
| Islets vs. Islets + NPs + Cyt | 12.50259**•** | 17.98871**•** | 22.75097**•** | 3.086029 |
| Islets vs. ASCs + Islets + Cyt | 11.24605**•** | 20.55891**•** | 23.38837**•** | 4.135233**•** |
| Islets vs. ASCs + Islets + NPs + Cyt | 13.45860**•** | 22.23310**•** | 31.47748**•** | 7.269424**•** |

**•** *Indicates statistically significant difference respect untreated control (p < 0.05)*

**Table S6.** Mean difference for inflammatory cytokines-associated gene expression relative to untreated human ASCs.

|  | ***Gene (human ASCs)*** | | | |
| --- | --- | --- | --- | --- |
| ***Comparison*** | ***TNF-α*** | ***IL-6*** | ***IL-1β*** | ***IL-15*** |
| ASCs vs. ASCs + NPs | 1.294792 | 1.025298 | 1.542055 | 1.335058 |
| ASCs vs. ASCs + Islets | 2.310468 | 5.233594**•** | 2.252164 | 1.786899 |
| ASCs vs. ASCs + Islets + NPs | 1.473669 | 5.22998**•** | 3.549768 | 1.043086 |
| ASCs vs. ASCs + Cyt | 105.1962**•** | 36.30781**•** | 389.0451**•** | 11.77606**•** |
| ASCs vs. ASCs + NPs + Cyt | 83.75293**•** | 30.83188**•** | 820.3515**•** | 13.61445**•** |
| ASCs vs. ASCs + Islets + Cyt | 78.34296**•** | 25.76321**•** | 498.8845**•** | 14.19058**•** |
| ASCs vs. ASCs + Islets + NPs + Cyt | 65.76578**•** | 29.92265**•** | 877.0008**•** | 13.89953**•** |

**•** *Indicates statistically significant difference respect untreated control (p < 0.05)*

**Table S7.** Mean difference for inflammatory cytokines-associated gene expression relative to untreated rat islets.

|  | ***Gene (Rat islets)*** | | | | |
| --- | --- | --- | --- | --- | --- |
| ***Comparison*** | ***TNF-α*** | | ***IL-6*** | ***IL-1β*** | ***IL-15*** |
| Islets vs. Islets + NPs | 1.332907 | | 1.005711 | 1.141563 | 1.182687 |
| Islets vs. ASCs + Islets | 2.198366 | | 1.685388 | 2.06538 | 1.283512 |
| Islets vs. ASCs + Islets + NPs | 1.401845 | | 1.363641 | 2.215134 | 1.526863 |
| Islets vs. Islets + Cyt | 8.081649 | | 3.347341 | 3.554676 | 2.530463 |
| Islets vs. Islets + NPs + Cyt | 7.488589 | | 1.708048 | 2.792544 | 2.624219 |
| Islets vs. ASCs + Islets + Cyt | 7.189522 | | 4.389348**•** | 9.862795**•** | 3.243396 |
| Islets vs. ASCs + Islets + NPs + Cyt | | 7.138386 | 2.353965 | 4.075679 | 1.579065 |

**•** *Indicates statistically significant difference respect untreated control (p < 0.05)*

**Table S8.** Mean difference for inflammatory cytokines-associated gene expression relative to untreated rat ASCs.

|  | ***Gene (Rat ASCs)*** | | | |
| --- | --- | --- | --- | --- |
| ***Comparison*** | ***TNF-α*** | ***IL-6*** | ***IL-1β*** | ***IL-15*** |
| ASCs vs. ASCs + NPs | 1.085325 | 1.544899 | 2.349633 | 1.199416 |
| ASCs vs. ASCs + Islets | 1.304368 | 1.28736 | 1.395083 | 1.348963 |
| ASCs vs. ASCs + Islets + NPs | 2.539803 | 1.048818 | 1.704512 | 1.040878 |
| ASCs vs. ASCs + Cyt | 17.02159**•** | 12.50259**•** | 118.5769**•** | 1.971061 |
| ASCs vs. ASCs + NPs + Cyt | 22.33572**•** | 9.660509**•** | 127.6439**•** | 1.091491 |
| ASCs vs. ASCs + Islets + Cyt | 11.61449**•** | 12.64736**•** | 123.3105**•** | 1.344002 |
| ASCs vs. ASCs + Islets + NPs + Cyt | 11.19438**•** | 11.72195**•** | 109.3956**•** | 1.208732 |

**•** *Indicates statistically significant difference respect untreated control (p < 0.05)*

**Table S9.** Mean difference for inflammatory chemokines-associated gene expression relative to untreated human islets.

|  | ***Gene (human islets)*** | | |
| --- | --- | --- | --- |
| ***Comparison*** | ***IL-8*** | ***MCP-1*** | ***IP-10*** |
| Islets vs. Islets + NPs | 1.08064 | 1.09989 | 1.29658 |
| Islets vs. ASCs + Islets | 1.21607 | 1.22242 | 1.94760 |
| Islets vs. ASCs + Islets + NPs | 1.01964 | 1.05249 | 1.17184 |
| Islets vs. Islets + Cyt | 19.49845**•** | 5.02805**•** | 6456.54229**•** |
| Islets vs. Islets + NPs + Cyt | 16.94338**•** | 4.22961 | 5847.90084**•** |
| Islets vs. ASCs + Islets + Cyt | 8.95159**•** | 4.19373 | 7888.60118**•** |
| Islets vs. ASCs + Islets + NPs + Cyt | 23.60478**•** | 5.98825**•** | 10715.19305**•** |

**•** *Indicates statistically significant difference respect untreated control (p < 0.05)*

**Table S10.** Mean difference for inflammatory chemokines-associated gene expression relative to untreated human ASCs.

|  | ***Gene (human ASCs)*** | | |
| --- | --- | --- | --- |
| ***Comparison*** | ***IL-8*** | ***MCP-1*** | ***IP-10*** |
| ASCs vs. ASCs + NPs | 1.31583066 | 1.50176 | 1.03531 |
| ASCs vs. ASCs + Islets | 8.36180432**•** | 4.56457**•** | 5.68722**•** |
| ASCs vs. ASCs + Islets + NPs | 7.59101763**•** | 4.06256**•** | 2.69650 |
| ASCs vs. ASCs + Cyt | 554.625713**•** | 31.91538**•** | 46131.75746**•** |
| ASCs vs. ASCs + NPs + Cyt | 831.763771**•** | 34.83373**•** | 45394.16167**•** |
| ASCs vs. ASCs + Islets + Cyt | 651.628394**•** | 31.26079**•** | 44771.33042**•** |
| ASCs vs. ASCs + Islets + NPs + Cyt | 924.698174**•** | 34.11929**•** | 43151.90768**•** |

**•** *Indicates statistically significant difference respect untreated control (p < 0.05)*

**Table S11.** Mean difference for inflammatory chemokines-associated gene expression relative to untreated rat islets.

|  | ***Gene (rat islets)*** | | |
| --- | --- | --- | --- |
| ***Comparison*** | ***CXCL-1*** | ***MCP-1*** | ***IP-10*** |
| Islets vs. Islets + NPs | 1.8454403 | 1.41351 | 1.07216 |
| Islets vs. ASCs + Islets | 1.56602972 | 1.42037 | 1.15952 |
| Islets vs. ASCs + Islets + NPs | 1.67224536 | 1.01950 | 1.12135 |
| Islets vs. Islets + Cyt | 3.12104478 | 10.66596**•** | 58.34451**•** |
| Islets vs. Islets + NPs + Cyt | 3.52046475 | 10.09253**•** | 28.37919**•** |
| Islets vs. ASCs + Islets + Cyt | 22.1819642**•** | 12.53141**•** | 57.14786**•** |
| Islets vs. ASCs + Islets + NPs + Cyt | 17.78279**•** | 10.02305**•** | 46.66594**•** |

**•** *Indicates statistically significant difference respect untreated control (p < 0.05)*

**Table S12.** Mean difference for inflammatory chemokines-associated gene expression relative to untreated rat ASCs.

|  | ***Gene (rat ASCs)*** | | |
| --- | --- | --- | --- |
| ***Comparison*** | ***CXCL-1*** | ***MCP-1*** | ***IP-10*** |
| ASCs vs. ASCs + NPs | 2.4299649 | 1.97333 | 1.41579 |
| ASCs vs. ASCs + Islets | 1.06944881 | 1.09792 | 2.10475 |
| ASCs vs. ASCs + Islets + NPs | 1.04030336 | 1.40799 | 2.97715 |
| ASCs vs. ASCs + Cyt | 28.5101827**•** | 6.20440**•** | 2517.67693**•** |
| ASCs vs. ASCs + NPs + Cyt | 32.2849412**•** | 8.97635**•** | 2172.70118**•** |
| ASCs vs. ASCs + Islets + Cyt | 22.9614865**•** | 7.33162**•** | 2471.72415**•** |
| ASCs vs. ASCs + Islets + NPs + Cyt | 28.0543364**•** | 8.42170**•** | 2172.70118**•** |

**•** *Indicates statistically significant difference respect untreated control (p < 0.05)*

**Table S13.** Mean difference for cell death-associated gene expression relative to untreated human ASCs.

|  | ***Gene (human ASC)*** | | | | | |
| --- | --- | --- | --- | --- | --- | --- |
| ***Comparison*** | ***RIPK1*** | ***RIPK3*** | ***BAD*** | ***BID*** | ***Caspase 3*** | ***Caspase 9*** |
| ASCs vs. ASCs + NPs | 1.51008 | 1.95704 | 1.22857 | 1.04486 | 1.432188 | 1.12873 |
| ASCs vs. ASCs + Islets | 1.344002 | 1.35800 | 1.48799 | 1.31492 | 3.006076 | 1.067112 |
| ASCs vs. ASCs + Islets + NPs | 1.683449 | 1.64286 | 1.29599 | 1.11792 | 1.245661 | 1.13802 |
| ASCs vs. ASCs + Cyt | 1.360818 | 6.35477• | 3.62410 | 4.21405• | 1.587084 | 1.118716 |
| ASCs vs. ASCs + NPs + Cyt | 2.65033 | 3.78443• | 1.31160 | 4.43507• | 2.602556 | 1.375942 |
| ASCs vs. ASCs + Islets + Cyt | 2.04597 | 3.79490• | 1.26940 | 3.82737• | 2.167704 | 1.157471 |
| ASCs vs. ASCs + Islets + NPs + Cyt | 1.60694 | 6.49531• | 2.79126 | 3.95367• | 2.740312 | 1.49039 |

**•** *Indicates statistically significant difference respect untreated control (p < 0.05)*
